# Field site selection: getting it right first time around

**DOI:** 10.1186/1475-2875-8-S2-S9

**Published:** 2009-11-16

**Authors:** Colin A Malcolm, Badria El Sayed, Ahmed Babiker, Romain Girod, Didier Fontenille, Bart GJ Knols, Abdel Hameed Nugud, Mark Q Benedict

**Affiliations:** 1 School of Biological and Chemical Sciences, Queen Mary, University of London, Mile End Road, London E1 4NS, UK; 2 Tropical Medicine Research Institute, National Centre for Research. P.O. Box 1304, Khartoum, Sudan; 3 National Centre for Research, Ministry of Science and Technology P.O. Box 2404, Khartoum, Sudan; 4 Institut Pasteur de la Guyane, Unité d'entomologie médicale, B.P. 6010, 97306 Cayenne Cedex, Guyane Française; 5 Institut de recherche pour le Développement, BP 64501, 34394 Montpellier Cedex 5, France; 6 Div. Infectious Diseases, Tropical Medicine & AIDS, Academic Medical Center, F4-217, Meibergdreef 9, 1105 AZ Amsterdam, The Netherlands and K&S Consulting, Kalkestraat 20, 6669 CP Dodewaard, The Netherlands; 7 National Health Laboratory, Ministry of Health, P.O. Box 1891, Khartoum 11111, Sudan; 8 International Atomic Energy Agency, Agency's Laboratories, Seibersdorf, A-2444, Austria

## Abstract

The selection of suitable field sites for integrated control of *Anopheles *mosquitoes using the sterile insect technique (SIT) requires consideration of the full gamut of factors facing most proposed control strategies, but four criteria identify an ideal site: 1) a single malaria vector, 2) an unstructured, relatively low density target population, 3) isolation of the target population and 4) actual or potential malaria incidence. Such a site can exist in a diverse range of situations or can be created. Two contrasting SIT field sites are examined here: the desert-flanked Dongola Reach of the Nile River in Northern State, Sudan, where malaria is endemic, and the island of La Reunion, where autochthonous malaria is rare but risk is persistent. The single malaria-transmitting vector at both sites is *Anopheles arabiensis*. In Sudan, the target area is a narrow 500 km corridor stretching from the rocky terrain at the Fourth Cataract - just above the new Merowe Dam, to the northernmost edge of the species range, close to Egypt. Vector distribution and temporal changes in density depend on the Nile level, ambient temperature and human activities. On La Reunion, the *An. arabiensis *population is coastal, limited and divided into three areas by altitude and exposure to the trade winds on the east coast. Mosquito vectors for other diseases are an issue at both sites, but of primary importance on La Reunion due to the recent chikungunya epidemic. The similarities and differences between these two sites in terms of suitability are discussed in the context of area-wide integrated vector management incorporating the SIT.

## Background

The Sterile Insect Technique (SIT) is an option for inclusion in area-wide integrated vector management (AW-IVM) programmes, where and when the necessary criteria have been met and it is the best of the alternatives. For any control method, but in particular methods based on mass release, field site issues, such as difficult and extensive terrain, complex local vector bionomics and poor infrastructure, can necessitate reliance on broad assumptions and extrapolation, when a critical factor may lie in specific details.

In contrast to efforts in the mid-1900s, modern evaluation of field sites benefits from substantial advances in technology and knowledge: Global positioning systems, geographic information systems and high resolution satellite images allow comprehensive storage and analysis of temporally and spatially referenced data relevant to vector bionomics and environmental variables [1]. Molecular techniques and genome project data greatly facilitate analysis of vector population genetics and insecticide resistance [2].

This paper examines two contrasting field sites where studies to develop AW-IVM including SIT are underway for controlling the malaria vector *Anopheles arabiensis*: the desert-isolated Dongola Reach of the Nile River in Northern State, Sudan and the French island of La Reunion in the Indian Ocean. While the uses of SIT at the two field sites are described as feasibility studies, it is not the technique itself that is in question, but rather the ability to surmount the successive practical and technical challenges of creating and sustaining an operational programme on a meaningful scale.

The appropriateness of SIT for use at each field site is a separate consideration. This chapter focuses on field site evaluation leading to the choice of SIT, rather than whether or not it can be successfully implemented. Furthermore much of the evaluation of a field site is general purpose when it comes to malaria control, so while factors such as capacity building, existing entomological information and national support are important considerations, the focus here is only on the factors most pertinent to SIT.

The problem was presented to a panel of experts at a meeting sponsored by the IAEA in Vienna in June, 2001 [3]. The panel recommended four primary criteria upon which to base the choice of sites at which SIT might be employed: (1) an isolated vector population, (2) one vector species, (3) a low density vector population amenable to inundation with released males, and (4) significant actual or potential malaria transmission.

Each of the criteria will be discussed below and considered in depth in relation to the field sites in Sudan and La Reunion. The criteria are a simple primary guide that illustrate an ideal situation, but a consideration of past studies and the two detailed examples will illustrate that disparate situations can be suitable for the development of SIT for use in the control of anophelines. Furthermore as SIT is used in the context of AW-IVM, the criteria of isolation and low density may be met through other control methods. The scope to find additional suitable sites is therefore substantial.

## Discussion

### Isolation

Even a small influx of inseminated females can significantly counter the suppression achieved by the released sterile males [4,5]. In ideal field sites the target mosquito population is naturally isolated, or there is scope to attain isolation with artificial barriers. Past examples include elimination of *Anopheles albimanus *within one season from Lake Apastepeque in El Salvador, where isolation was promoted by the limited availability of larval breeding sites, bloodmeal hosts and shelter and by hills on two sides [6,7]. *Culex tarsalis *populations in a canyon in California [8] and *Culex quinquefasciatus *populations on islands off the coast of Florida [9,10] were well isolated, and programme failure and success, respectively, were not affected by immigration.

The first example of successful genetic control of mosquitoes illustrates how even temporary isolation may be exploited; *Cx. quinquefasciatus *was eliminated within one season from a small Myanmar village surrounded by rice fields that were completely dry in winter [11]. A current SIT programme in Italy to suppress *Aedes albopictus *benefits from the absence of suitable breeding sites in open farmland surrounding the target villages [12].

Isolation can be enhanced or created artificially by source reduction, insecticide application and land use changes. An insecticide barrier was used to isolate a *Cx. quinquefasciatus *population in two villages south of New Delhi [13] and to complete the isolation of the *An. albimanus *population in the second release programme in El Salvador where the Pacific coast and a mountain range provided the primary barriers [14]. Tyre dumps in New Delhi provided relatively isolated foci of *Aedes aegypti *[15]. Releases targeting a population of *Anopheles gambiae *in the small village of Pala, near Bob-Dioulasso in Burkina Faso, relied on distance from neighbouring villages [16]. Slightly different was a *Culex pipiens *population isolated in a subterranean well in a small village near Montpellier, France [17]. The importance of isolation and the necessity for a comprehensive and detailed field site evaluation was illustrated in the second programme in El Salvador, where the contribution of *An. albimanus *larval breeding sites forming at the mouths of drying up rivers (*esteros*) was not accounted for (P. Kaiser, pers. comm.) and immigration into the release area exceeded expectations [14].

The need for isolation can limit the scope of SIT, but the above examples indicate the diverse nature of the sites targeted in the past. Table 1 attempts an overview of the potential to find or create an appropriate target population. Combinations of the factors listed further enhance isolation, but the complexity may require detailed analysis to unveil the suitability of the site. The following sections describe how isolation exists or can be created in the two specific sites. Isolating factors that will be identified are related to geography, climate, mosquito biology and human activities.

**Table 1:** Examples of naturally occurring, unintentional and deliberately created human made factors that may promote isolation

	**Natural isolating factors**	**Human activity with incidental effects**	**Artificial and deliberate methods**
Physical barriers	Sea, large rivers and lakes, mountains and hills, desert and barren or poor terrain	Buildings, roads, railways	Insecticide, drainage
Vegetation	Unsuitable habitat type, excessive shade, no shelter	Large monospecies plantations	Land use change e. g. clearing and planting
Distance	Between suitable habitats, to nearest host	Sparse human presence, no farm animals, no plumbing or irrigation	Zones with no host, no natural or human-made breeding sites etc.
Time	Seasonal events, flood, animal bird migration	Crops, animal movement	Time limited or interrupted water supplies
Temperature	Seasonal changes, latitude, altitude		
Competition	Neighbouring habitat zones favour a competitor		Promotion of benign competitor or predator

### The Northern State of Sudan

The Republic of Sudan is the largest country in Africa, covering 2.5 million km^2^. Northern State, located in the north and bordered by Libya, Egypt and Chad, occupies nearly one fifth of the country and is the largest of the 26 states, yet has the lowest population density (~567,000). It is very arid and almost entirely desert: rainfall is rare, whereas dust storms are common. Temperatures reach 47°C in summer and as low as 7°C at night in the winter.

**Figure 1:**
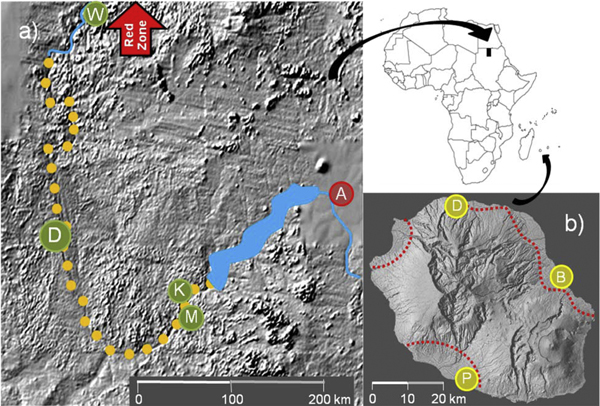
**Maps of the field sites**. (a) The Nile valley in northern Sudan. Yellow dots indicate the field site extending from the Merowe Dam lake (blue) to the *An. arabiensis*-free Red Zone. In Northern State: D - Dongola, K - Kareima, M - Merowe, W-Wadi Halfa in Nile State: A-Abu Hamad. (b) La Reunion. Dotted red lines indicated the inland boundary of three coastal populations of *An. arabiensis*. D-Saint Denise, P-Saint Pierre and B-Saint Benoît.

The Dongola Reach, between the Third and Fourth Cataracts, accounts for about two thirds of the length of the Nile within Northern State (Figure 1a). Above the Fourth Cataract and below the Third Cataract, the bedrock is crystalline basement; there is little alluvial soil, little scope for agriculture and a correspondingly low human population density. Between the cataracts the river passes over mostly sandstone and is flanked by wide alluvial flood plains [18].

As all agriculture in this region is based on irrigation and the Nile flood, most of the State's people live alongside this stretch of the river, particularly within Dongola and Merowe provinces. Land under cultivation is more or less continuous along both banks varying in almost exact proportion with the human population density. Date palms predominate, while field crops are seasonal, being greater in winter when wheat and beans are grown, than in summer when maize and sorghum are common.

Travel to, and in, Northern State is slow and difficult. Most roads are fair-weather earth and sand tracks, asphalt or gravel. A few years ago less than one third were paved, but this is changing rapidly largely due to the Merowe Dam Project (described more fully below). There are railway lines from the south via Abu Hamed in neighbouring Nile State to Kareima on the eastern state border and Wadi Halfa near the border with Egypt, but these no longer provide passenger service. There is a scheduled domestic air transport service to Dongola from Khartoum, and there are other airfields including one at Merowe that is being improved for regular commercial traffic.

The Merowe Dam Project is the first stage of the largest development project in northern Sudan and is transforming the area. This hydroelectricity dam has been built on the Nile in the vicinity of Hamdab, north of Merowe. The reservoir is expected to cover an area of 711 km^2^. The project may be followed by construction of canals parallel to the river reaching as far as Dongola in a major expansion of irrigation-based agriculture.

The northern edge of the distribution of *An. arabiensis *is downriver beyond Dongola approximately 300 km south of the border with Egypt. It is kept in check by unsuitable terrain, low human population density, less favourable climate and the Gambiae Control Project (GCP), but occasional incursions of the mosquito as far as Wadi Halfa have been found.

Current mosquito control projects affect the suppression and isolation of the mosquito populations against which SIT is planned. They involve collaboration between the State Ministry of Health, the Malaria Administration and the GCP. The GCP is a joint protocol between the National Ministries of Health of Egypt and Sudan to maintain an *An. arabiensis*-free zone just south of the Egyptian border (which borders the SIT target area). Egyptian control activity in Sudan is a historical remnant of a major malaria epidemic in Egypt in the 1940s caused by the spread of *An. gambiae s.l. *(but almost certainly *An. arabiensis*) from Sudan. The mosquito was successfully eradicated, but could easily re-invade.

The current GCP was implemented in 1970 with the creation of three zones. The Red Zone, which extended north from Akasha, was considered to be free of *An. arabiensis*. The Yellow Zone to the south as far as Abri was the main focus of control operations. The Green Zone extended further south to Abu Fatma. The Red Zone has since been extended south to Abri, despite occasional discoveries of the vector. The Green Zone now encompasses Dongola.

In the past most control activities throughout the State involved local sanitation workers who removed or treated larval breeding sites. Larviciding and adulticiding was used extensively only in Dongola and further north where the GCP was operating. The Malaria Administration now has more direct involvement, and the GCP has expanded to cover the whole State. Nevertheless coverage is still very patchy, particularly upriver from Dongola.

Mosquitoes are restricted by the desert to precisely the areas in which people live. Immigration via the desert is impossible and passive transportation via roads is improbable. Our efforts to transport mosquitoes by car for periods longer than about half an hour were only successful if precautions were made to control temperature and, for adults, humidity. The width of the cultivated areas is only a few kilometres, which provides scope for the creation of control barriers that can be extended in a rolling carpet fashion up or downstream.

The few relatively isolated villages and the inhospitable terrain above the Fourth Cataract limit survival and movement of *An. arabiensis *along the Abu Hamad Reach, so the Northern State mosquito population is relatively isolated (R. Ashrag, pers. comm.). The isolation will become complete as a consequence of the Merowe Dam. The reservoir lake will stretch for about 200 km along the previous course of the Nile and extend out into harsh rocky terrain and desert unsuitable for agriculture and human habitation. It will eradicate any mosquitoes in the area and pose a significant barrier to mosquito dispersal downriver for many years to come, if not indefinitely,

The Nile Valley of Northern State therefore provides a field site no more than 500 km long and mostly less than 10 km wide that is well isolated by a combination of factors, but primarily the desert. The very limited opportunity for reinvasion through passive transportation can be addressed by modest surveillance and control efforts conducted some distance away at the start of the relatively few routes to the north.

### La Reunion

The island of La Reunion lies in the Indian Ocean, about 700 km from Madagascar and approximately 200 km southwest of Mauritius. La Reunion is 63 km long and covers 2,512 km^2^. The human population is close to 800,000 and most live in coastal areas. The island was formed from two volcanoes (the smaller one is still active) and almost three quarters of the land is above 500 m (Figure 1b). Three deep valleys were formed from collapsed sections of the larger volcano with further erosion by large rivers extending to the coast. There are about twelve permanent rivers and hundreds that are seasonal. All these features together create a rugged, high interior and a hilly to flat coastal ring.

The climate is mainly tropical, but varies with altitude and aspect. The east is exposed to the trade winds, which bring about 4-8 times more rainfall than in the west. The hot, wet season is from November to April when average temperatures are around 25°C and vary only a few degrees over a full day. Relative humidity remains high throughout the year, except in the west where it can reach as low as 50%. Cyclones in January and February are relatively common.

Sugar cane occupies about 50% of the cultivated land, extending across the coastal lowlands and lower slopes of the mountains and accounts for 69% of all exports. Tourism is important and was badly affected by the recent chikungunya epidemic [19]. About half of the 2,800 km of roads are paved. All areas where the vector is present can be visited in a matter of hours and certainly within a working day, although the specific breeding sites in river beds often must be reached by foot.

On La Reunion, leaving aside passive transportation, the overall isolation of the mosquito population is not in question. The total area of the island is less than the proposed control area in Sudan, and the area occupied by *An. arabiensis *is much smaller. There is again scope for a release programme to be carried out in stages as the vector is found within three distinct sectors covering about 100 km^2 ^in the northeast, about 50 km^2 ^in the northwest and about 30 km^2 ^in the southwest (Figure 1b). These are separated by mountain ridges or arid areas that extend down to the coast. If needed, these barriers could be artificially reinforced to ensure complete isolation.

Passive transportation of *An. arabiensis *onto the island and between release zones via human transportation is an important concern and will become more so as vector free areas develop. In contrast to northern Sudan, survival of adult mosquitoes in cars is likely and travelling times are short. The risk of malaria and vectors becoming re-established via infected mosquitoes and people arriving at sea and airports is currently tackled by monitoring and control operations. If a successful control programme using SIT leads to elimination of the mosquito population, this will become the primary focus of subsequent anti-malaria efforts.

### Single vector

While it is possible to target more than one vector through the simultaneous release of sterile males of different species, reducing malaria transmission by release of a single species will be more cost effective. Density dependent regulation of the target vector has been recognised as a problem in past studies, because low density larval populations can lead to higher adult numbers compared to crowded breeding sites [4]. This needs to be taken into account when release schedules and release numbers are being considered. Of equal concern is the risk that reduction or elimination of the target species may favour other vectors. It is also important to consider multiple diseases, not just multiple vectors for one disease. The vector population should be unstructured, since any forms, ecotypes, incipient or sibling species may vary in their capacity to mate with released males and in their contribution to disease transmission.

### Northern State

*Anopheles arabiensis *is the only anopheline vector in Northern State [20]. The most extensive genetic study of this species in Sudan was based on the analysis of chromosome inversion polymorphism which indicated a fairly uniform population, although with quite a high frequency of inversion 2R in Kashmel Girba in Eastern Sudan [21]. Samples from Northern State were too limited to draw strong conclusions. A study based on microsatellite DNA polymorphism covered only two locations in Eastern Sudan, but concluded that in a transect from Sudan to Mozambique, *An. arabiensis *showed significant differentiation between populations separated by more than 200 km [22]. Therefore, there was evidence of restricted gene flow, which in Sudan at least, could be the consequence of geographical features separating the populations.

The presence of several other anophelines has been reported, i.e. *Anopheles rufipes*, *Anopheles pharoensis *and *Anopheles multicolour*, but all records of these species were made over fifty years ago and they have not been found since [23]. From the standpoint of environmental effects of interspecific interactions, it is possible that removal of *An. arabiensis *would affect the density of culicine species since their larval development sites overlap to some extent [24]. There is no evidence of other mosquito-borne diseases in Northern State, but vectors are present for filariasis, which is a major problem in neighbouring Egypt.

### La Reunion

The broadest collection of information regarding anophelines on La Reunion has been collected and synthesised by Girod [25]. Based on historical information about malaria transmission and immigration, he hypothesised that *An. arabiensis *was introduced to Reunion from Mauritius during a cyclone in the mid 1800s. *Anopheles arabiensis *is the only malaria vector on Reunion Island [26]. Studies of *An. arabiensis *over large geographical distances on mainland Africa show differentiation, but as yet no published reports of distinct forms [27]. Only one other anopheline is found - *Anopheles coustani *- which does bite humans, but is not considered a potential vector of malaria on the island [25].

A comparison of microsatellite DNA polymorphism in a sample of *An. arabiensis *from La Reunion with samples from neighbouring islands and West Africa [27], and an analysis of chromosomal inversion polymorphism [28] produced results consistent with expectations for a small quite isolated population.

Past malaria control campaigns, heavily reliant on indoor insecticide spraying, have selected this mosquito to become exophilic and exophagic [29]. The available data suggest it is now mainly zoophilic, but opportunistic, so that human biting will increase where facilitated by proximity.

Despite the isolation and specific selection pressures, crossing studies between laboratory strains originally from mainland locations and La Reunion mosquitoes showed no evidence of incompatibility [28]. The population is divided geographically, but this is unlikely to have led to differentiation since originally the population extended across most of the island at a much higher density and the reduction to three relatively isolated foci is the consequence of control efforts over the last sixty years.

### Population dynamics

To attain and sustain the optimal release of sterile males it is vital to have good data on the target mosquito population density and distribution throughout the year and in response to changes in climate or other major factors such as the Nile flood in Northern State. In contrast to insecticide, SIT increases in efficiency as the density of the target population decreases, so SIT is a good option when the starting point is a low-density population. If the starting point is high density, then SIT will be cost effective at a later stage. This was illustrated by the eradication of the tsetse fly, *Glossina austeni*, from Unguja Island, where the population was suppressed with pyrethroids prior to using SIT [30].

An uneven mosquito distribution, especially with poor roads, limited or inaccessible terrain and long distances, may also favour the choice of SIT over insecticide in an area-wide approach. Both of the field sites described here illustrate the above points. In each case the target population is low density. The delivery of insecticide to the vector is hindered in Northern Sudan by long distances and difficult terrain and in La Reunion by a change in the vector's behaviour from endophilic to exophilic.

### Northern State, Sudan

The temporal and spatial changes in *An. arabiensis *density are mainly determined by the flooding of the Nile and changes in ambient temperature [20]. The Nile reaches its highest levels in September coinciding with the lowest density of *An. arabiensis*. As the river level falls, suitable breeding sites form, but low winter temperatures delay the resurgence in mosquito numbers. By March, the river level is down almost to its minimum, but the temperature has not yet reached the summer maxima resulting in the peak of *An. arabiensis *density. A month later the temperatures are much higher and mosquito numbers drop to an intermediate level which is mostly sustained throughout the summer until the flood [20,24].

The location, relative timing and magnitude of these events may be critical, since past studies in Upper Egypt record quite a different pattern [31]. An early flood, perhaps of less magnitude and a late or less severe winter, could conceivably produce a peak density in late October and early November. Much depends on whether the primary larval breeding sites are riverside or inland.

Extensive studies of larval development sites have been completed and provide a valuable source of detailed information about their temporal and spatial characteristics [24]. The riverside can be divided into three zones and within these zones the presence or absence of larval breeding sites can be associated with particular features. *Anopheles arabiensis *was most reliably found in riverbank pools surrounded by grassy vegetation. The low-lying grassy banks are undulating, so pools form and slowly dry up as the river level drops. Some may remain throughout the summer, particularly if they are connected to the river underground, or are deliberately maintained to facilitate irrigation. These are common, but there are also long stretches of very steep river bank, where *An. arabiensis *larvae are almost never found. All other breeding sites are the consequence of human activity, usually associated with poor maintenance and distribution of water supply; however these are also influenced by seasonal factors and by location. In particular, water conservation, such as irrigation of fields in rotation and limiting water supplies to houses for part of the day, is more extensive in summer months. Small villages may have very little plumbing, less reliable water supplies and many houses may be uninhabited, whereas in places like Dongola, Merowe and Kareima the number of larval breeding sites associated with leaking pipes is more likely to correlate with the size of residential areas.

There is also more agriculture associated with the larger urban conurbations, with more substantial and reliable canals extending much further from the river. Due to water flow, conservation and high temperatures the irrigation channels rarely provide larval breeding sites, even where run-off pools form from poor maintenance. Occasionally, larvae are found in pools that form from leaking water pumps that supply the channels. In both cases the water is usually dirty and unsuitable for *An. arabiensis. *However, there is enough scope for *An. arabiensis *to continue breeding throughout the year, and the available vector density and malaria incidence data indicate that this is the case. It is unlikely that aestivation occurs [20,31], although the possibility cannot yet be ruled out.

### La Reunion

While the Sudan analysis is based on recent surveys, the data for La Reunion is temporally extensive and spatially detailed. As of 1985, all actual and potential breeding sites surveyed have been placed into six categories with spatial and temporal references and entered in a geographical information system. The main breeding sites are partially dry river beds and puddles formed from wheel ruts in the sugar cane fields. There are no breeding sites found above 600 m, although these did exist in the past when the mosquito population was much larger. *Anopheles arabiensis *breeds throughout the year, with a slightly lower density occurring during the cooler dry season.

### Malaria

#### Northern State

Malaria in Northern State appears to occur throughout most of the year with a peak incidence in May or June, but most of the available data are based only on admissions to the 26 clinics and hospitals. These records are not detailed, nor necessarily reliable due to a lack of robust diagnosis, and all locations do not follow a predictable pattern. Wadi Halfa hospital is particularly notable, since it has malaria admissions in similar proportions to other hospitals, yet it is in an area where the vector is not present. This could be explained by large numbers of travellers en route to and from Egypt and the exchange of visits with relatives in New Halfa. At other hospitals, unusual peaks in the data could be due to an influx of casual labour for harvests or development projects. At present, it is difficult to assess the amount of malaria transmission actually occurring in Northern State; that it is occurring and that it is significant is not in doubt [32].

The lack of detailed data on malaria case histories in Northern State means there is only anecdotal information on the location of high-risk areas. For example it is not possible to assess if risk is greater in residential areas close to the riverbank surrounded by fields or palm groves than those further away surrounded by desert. There is also no published information on dispersal of the vector so one activity of the National team is to perform mark-release-recapture measurements, the data from which are now being collected. Another part of the National effort to support preparation for SIT is to perform more extensive malaria surveys and to use better diagnostic procedures and equipment, and to provide training in the clinics and hospitals.

From a national perspective, malaria control in northern Sudan requires as much if not more resource input than in other areas to tackle a far less severe problem. It is nevertheless an important area for the national economy and one with considerable potential for further investment and development. From a regional perspective malaria in northern Sudan and Northern State in particular, is also important, because it poses a threat to Egypt and Libya.

#### La Reunion

On La Reunion malaria was once well established across much of the island, particularly in urban areas [33]. A control campaign using mainly DDT for spraying inside houses and larval breeding sites, combined with chemical prophylaxis distributed via schools, was started in 1948 and produced a significant impact. A concerted programme to eradicate malaria was started in the 1960s and by the 1970s nearly all malaria cases were imported. Eradication was formally recognised by WHO early in 1979 [33].

It is, therefore, now more than 25 years since the residents of La Reunion were routinely exposed to malaria infection, and only rarely do autochthonous cases occur. Since up to 170 travellers infected with malaria arrive on the island every year, the risk of an outbreak is significant and could result in high mortality, especially as the predominant infection is with *Plasmodium falciparum. *Moreover, the continuous significant expenditures on vector surveillance and control increase the attractiveness of an SIT programme, particularly if it resulted in elimination. Annual expenditures prior to the modern chikungunya epidemics were estimated at € 3,350,000 per annum, 70% of which was dedicated to vector control [25]. A vector surveillance programme would still be necessary for the detection and control of passively transported mosquitoes to insure against re-invasion, but on a much more modest scale.

The current anti-malaria campaign is carried out by La Direction Régionale des Affaires Sanitaires et Sociales. It involves the monitoring of all travellers from known malarious regions with a free service of blood tests and advice on appropriate treatment. Abate^® ^is applied to positive and potential larval breeding sites in the vicinity of the harbour and airports with additional adulticiding in and around the houses of definite, or likely, malaria cases.

## Other key issues for SIT development

The facilities for sterile male production for Northern State will be in Khartoum, close to an existing radiation source and the scientific institutions with the necessary expertise and trained technical staff needed to support the programme and where supplies and maintenance for the building, equipment and utilities will be more reliable. This will entail a four- to six-hour overland journey, all of it through the desert and at high ambient temperatures most of the year. Transport by air is feasible and may also be favoured for releases, although this has never been done with mosquitoes. The river is another option for the distribution from a delivery centre to release points and likely to be the most practical. This concept echoes an old idea: in 1907, the Wellcome Research Laboratories based in Khartoum, outfitted a boat called "Culex Pipiens" as a floating laboratory to overcome the many problems of field work and this approach has also been adopted by the GCP in Egypt.

La Reunion has better infrastructure for weather monitoring and prediction and both local and international communications. Expertise on insect pests and research facilities are well established on the island, whereas in Sudan most relevant expertise and facilities are based in Khartoum, although research on agriculturally important pests is reasonably well served within Northern State.

Capability strengthening is critical and must be considered at all levels, even retraining the most unskilled to gather new useful information. On La Reunion the obvious solution is that those involved with the current control programme will take the principal role in developing and implementing field operations. In Sudan, this is much more complex, the current control effort in Northern State involves three organisations and the development and delivery of the SIT project will involve at least another six. All efforts must be made to eliminate unnecessary duplication of effort, competition and misunderstanding.

### The unexpected

There are no safeguards against unexpected events that could have a dramatic impact on a release programme, but it does help to identify known risks. The occurrence of cyclones on La Reunion is an obvious example; these can bring very heavy rainfall and damaging winds. Sandstorms are a similar problem in Northern State. Less frequent and equally irregular are flash floods, very high river floods due to heavy rainfall in the Ethiopian Mountains and breached flood defences. These affected Northern State in 1988, 1994, 1998 and 1999. In 1998, 28,000 people were left homeless. In 1999 the riverbank south of Dongola broke, flooding 90% of the town and destroying five villages [34]. These events peaked in September when the temperatures were still relatively high, with an inevitable increase in disease incidence including malaria. Less conspicuous changes in timing of the flood, flood level and the onset of winter could also produce unexpected peaks in mosquito density. The creation of larval breeding sites through poor maintenance of water channels, pipes, taps, pumps and wells also has an element of unpredictability.

### Key issues

• Containment of release zones will require monitoring of transport, development projects, seasonal traffic and research on natural and artificial barriers.

• Other control measures should be integrated with SIT as standard, but in addition consideration is needed for initial population suppression, artificial barriers, control of other vectors and as part of contingency plans in the event of a breakdown in the SIT programme.

• Assessment and periodic reassessment of single vector status versus its population structure, secondary malaria vectors and vectors of other diseases.

• Population size, density and distribution in relation to terrain, climate and river levels, nature of larval breeding sites and human influence, vector behaviour and physiology.

• Operational logistics of transport and release of sterile males; organisation, capability strengthening and training for SIT and integration with existing vector control.

• Malaria incidence, geographic origin and cost.

• Unpredictable events, at least in the long-term, such as flash floods, sandstorms and cyclones.

## Conclusion

While both of these two field sites meet the criteria for SIT, they could not be more different. For vector control, La Reunion has some advantages compared with Northern State in terms of size and accessibility, in infrastructure, resources and technology and in the existing knowledge base for vector bionomics. However, the impact on malaria in Sudan will be greater, the reduction in risk to neighbouring countries substantial and an area with exciting development prospects of national importance will be freed of a significant hindrance to progress.

Some people might be tempted to conclude that areas such as these are unique, and that even if SIT is proven to be a valuable component of AW-IVM, it will be restricted to relatively unimportant status because its application will be limited to such unusual locations. SIT is, of course, not a stand-alone, off-the-shelf generic tool for mosquito control, but the diversity of the two sites considered provides an indication of just how different suitable field sites can be. When the range of field sites in related studies several decades ago is considered (Table 1), it is not difficult to envisage a wide and diverse range of potentially suitable field sites. Many may only be unveiled as such after field site evaluation and consideration of SIT alongside other components of the overall control strategy.

From another perspective, the projects described here are small and will start very small, but success in Northern State could see an extension of the programme to neighbouring River Nile State and perhaps even to Khartoum and its environs. Success on La Reunion could see an extension to other islands in the Indian Ocean. The long-term prospect is a very large area not only free of malaria, but free from the risk of a return of malaria.

## Competing interests

The authors declare that they have no competing interests.

## Authors' contributions

C. Malcolm composed most of the text and summarized many of his personal observations regarding Sudan. The remaining authors reviewed and approved the content.
